# A simplified electrochemical model for lithium-ion batteries based on ensemble learning

**DOI:** 10.1016/j.isci.2024.109685

**Published:** 2024-04-10

**Authors:** Guorong Zhu, Chun Kong, Jing V. Wang, Weihua Chen, Qian Wang, Jianqiang Kang

**Affiliations:** 1 School of Automation, Wuhan University of Technology, Wuhan 430070, P.R. China; 2 Hubei Key Laboratory of Advanced Technology for Automotive Components, Wuhan University of Technology, Wuhan 430070, P.R. China; 3 Hubei Collaborative Innovation Center for Automotive Components Technology, Wuhan 430070, P.R. China; 4 College of Chemistry & Green Catalysis Center, Zhengzhou University, Zhengzhou 450001, P.R. China

**Keywords:** Electrochemistry, Energy systems

## Abstract

The mass transfer in lithium-ion batteries is a low-frequency dynamic that affects their voltage and performance. To find an effective way to describe the mass transfer in lithium-ion batteries, a simplified electrochemical lithium-ion battery model based on ensemble learning is proposed. The proposed model simplifies lithium-ion transfer in electrode particles with ensemble learning which ensembles discrete-time realization algorithm (DRA), fractional-order Padé approximation model (FOM), and three parameters (TPM) parabolic. The lithium-ion transfer in the electrolyte is simplified by the first-order inertial element (FIE). The results show that the proposed model achieves not only accurate lithium-ion concentration prediction in solid and electrolyte phase but also precise voltage prediction with low computational complexity.

## Highlights

• A simplified electrochemical lithium-ion batteries model based on ensemble learning
• Solid phase diffusion is simplified by the ensemble learning model
• Electrolyte phase diffusion is simplified by the first-order inertial element
• The proposed model achieves precise voltage and internal state prediction

## Graphical abstract

**Figure:**
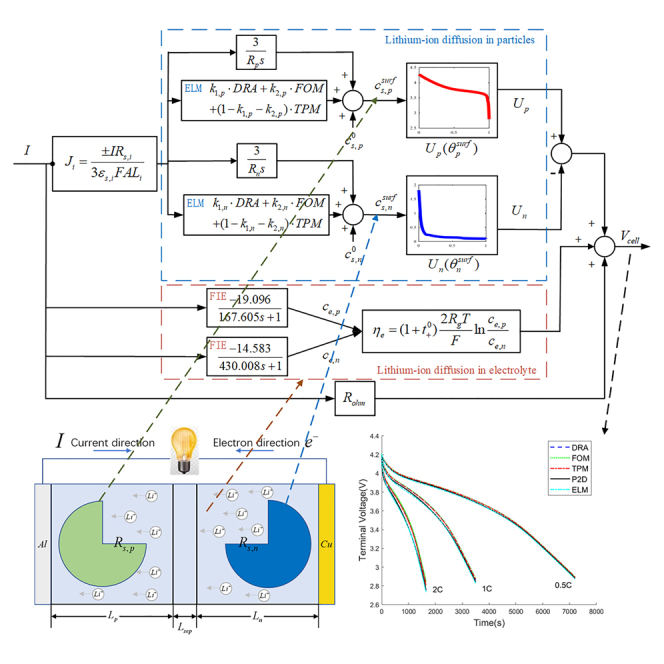

## Introduction

With the increasing depletion of fossil energy and the emission of greenhouse gases, it is urgent to electrify transportation widely. Developing electric vehicles (EVs) is one of the most important parts of transportation electrification.^1^^,^^2^ The battery pack is the power source of EVs and is their most expensive part. An accurate electrochemical battery model with low computational complexity is needed to monitor the critical states of batteries, maintain the safe and stable operation of batteries, and maximize batteries’ performance and service life.^3^

An accurate lithium-ion battery model is the key to achieve accurate battery state estimation. The equivalent circuit model (ECM) is a classical and commonly used lithium-ion batteries model with low computational complexity. Plett^4^^,^^5^^,^^6^ uses an extended Kalman filter (EKF) based on ECM to estimate the desired internal states. However, ECM cannot achieve high-precision prediction and describe the internal electrochemical mechanism of batteries, which limits monitoring high-precision multistate internal states in real-time embedded systems. Physics-based lithium-ion battery models can accurately describe the internal mechanism of batteries. The Pseudo-2D (P2D) model proposed by Newman et al.^7^^,^^8^ is a well-known physics-based electrochemical model, which consists of four coupled partial differential equations (PDEs) and one algebraic equation. The four PDEs govern the basic physical principles of mass and charge conservation in solid and electrolyte materials of the cell, and the algebraic equation describes the lithium-ion flux between solid material and electrolyte material.^9^ However, these PDEs are computationally complex, which stems the P2D model from being applied in real-time embedded systems.^10^ To reduce the computational complexity of the P2D model, a single particle (SP) model is proposed.^11^ In the SP model, the cell electrode is regarded as a single particle, and it is assumed that lithium-ions only diffuse in the electrode particle radial direction. The SP model ignores the electrolyte phase lithium-ion distribution and considers the electrolyte phase potential as a constant.^12^ Although the SP model significantly simplifies the physical mechanism of lithium-ion batteries, there are still solid phase mass and charge conservation PDEs in the SP model, which is hard for embedded systems to solve. Therefore, it is necessary to simplify and approximate these PDEs. Han et al.^13^^,^^14^ simplified the difference between the electrode surface lithium-ion concentration and the mean lithium-ion concentration by a system which consists of several first-order processes. The approximation system achieves highly accurate voltage prediction, but its parameters need to be identified for different cells. Lee et al.^15^ used the discrete-time realization algorithm (DRA) to find a reduced-order discrete-time realization of the infinite-order transcendental transfer function which describes the solid phase lithium-ion distribution. Guo et al.^16^ proposed a physics-based fractional-order model which simplifies the solid phase diffusion process by the fractional-order Padé approximation method (FOM). Luo et al.^17^ developed a simplified electrochemical model with the three parameters approximation method (TPM) to describe the solid phase diffusion process. For further improving the accuracy of the electrochemical lithium-ion batteries model, especially when the cell charges and discharges at high rates of current, the lithium-ion transfer in the electrolyte is modeled and added to SP model. Moura et al.^18^ derived an electrolyte observer for SP model, which estimates the lithium-ion distribution in the electrolyte. Zhu et al.^19^ considered polarization in the electrolyte with a two-state system, and achieved precise voltage prediction.

For further improving the accuracy and robustness of lithium-ion batteries models, machine learning methods have been used in the modeling and application of lithium-ion batteries. Shi et al.^20^ established a physics-informed machine learning method to model the degradation characteristics of lithium-ion batteries, and achieves accurate remaining useful life prediction. Zhang et al.^21^ proposed a distinctive method for solid-state lithium ion conductors’ discovery with unsupervised learning. They discovers 16 new fast Li-conductors with conductivities of $10^{-4}∼10^{-1}$ S/cm. Ensemble learning is a typical machine learning technique that combines multiple models to achieve better predictive performance than any of the individual models alone.^22^ Zhang et al.^23^ proposed a stacking ensemble learning paradigm for the state of health estimation of lithium-ion batteries. Their paradigm increases adaptability to different features by using base learners with different structures and achieves accurate state of health estimation under different working conditions. To achieve accurate state of charge estimation, Zhao et al.^24^ developed an improved adaptive boosting algorithm which ensembles extreme learning machines. Their model has good robustness and accuracy in state of charge estimation of lithium-ion batteries under various working conditions. Ma et al.^25^ developed a multi-view feature fusion method based on a support vector regression ensemble strategy to improve the performance of fusing multiple extracted degradation features of lithium-ion batteries, which improves the performance of lithium-ion battery SOH predictors.

Mass transfer is a low-frequency dynamic in lithium-ion batteries, which dominates the electrochemical reaction rate in lithium-ion batteries, and determines the voltage of lithium-ion batteries. The key to lithium-ion batteries modeling is the modeling of mass transfer in lithium-ion batteries. Therefore, a simplified electrochemical lithium-ion batteries model with ensemble learning is proposed to simplify the lithium-ion transfer in electrode particles and electrolytes. The original contributions of the proposed model are summarized as follows.

a) The mathematical principles of the simplified electrochemical model are derived and analyzed.
b) An ensemble learning model, which ensembles DRA, FOM, and TPM, is proposed to simplify lithium-ion transfer in electrode particles.
c) A first-order inertial element (FIE) is proposed to simplify lithium-ion transfer in the electrolyte.

This paper is organized as follows. Simplified electrochemical model section introduces the basic assumptions of the simplified electrochemical model, and derives the mathematical equations of the simplified electrochemical model based on physics mechanism. Ensemble learning model section derives and models the simplified electrochemical model based on ensemble learning. Simulation and model verification section compares the proposed model with DRA, FOM, TPM, and P2D, and verifies the accuracy and computational complexity of the proposed model. The conclusion is made in conclusions section.

### Simplified electrochemical model

#### Model structure

To accelerate the computational speed of electrochemical models and simplify the structure of traditional electrochemical models, a simplified electrochemical model for lithium-ion batteries is proposed, so that the electrochemical model can be applied to real-time BMS. There are some basic assumptions in the simplified electrochemical model.^12^

(1) The cell electrode is regarded as a single spherical particle, and the potentials at the same radial distance from the sphere center are equal.
(2) The solid phase diffusion is only considered in the radial direction.
(3) The lithium-ion pore wall flux is uniform in electrode particles.

**Figure 1:**
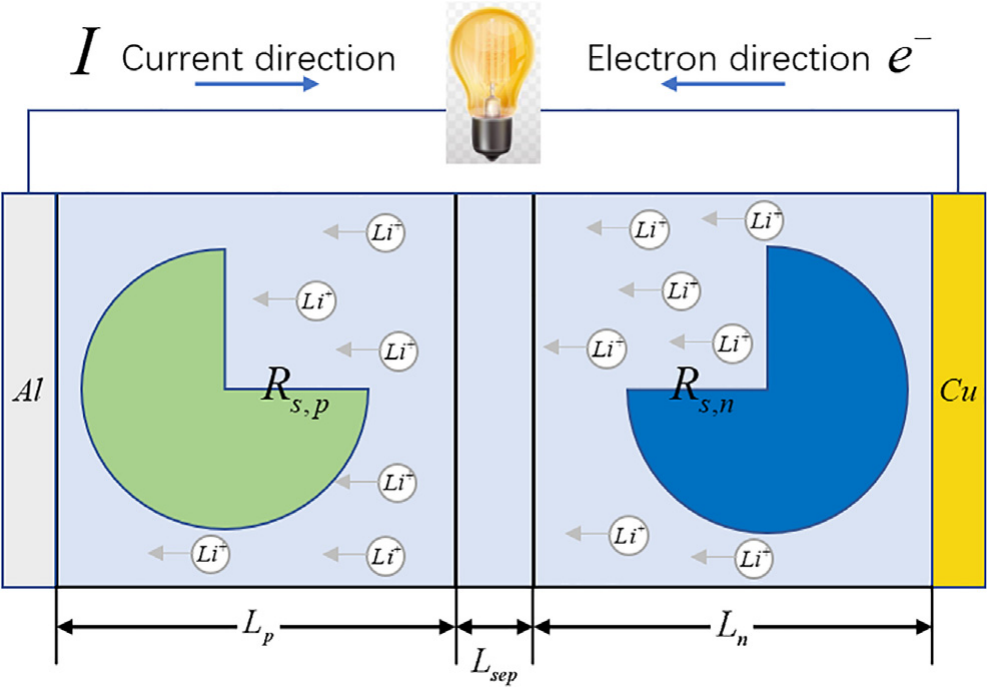
The structure of the single particle model

The structure of the simplified electrochemical model is shown in Figure 1. The simplified electrochemical model greatly simplifies traditional physics-based electrochemical models in terms of model structure. However, there are still infinite-order transcendental transfer functions in the simplified electrochemical model, which is too complex to be applied directly in an embedded system.

#### Model deviation

##### Open circuit voltage

The simplified electrochemical model is a typical sandwich structure, which mainly consists of a positive electrode, a separator, and a negative electrode. When a cell is discharging, lithium-ion flows from the negative electrode to the positive electrode, and the current flows from the aluminum current collector to the copper current collector through the load circuit. Hence, the lithium-ion pore wall flux $J_{i}$ can be calculated by the applied current *I* as^26^

(Equation 1) $$J_{i}=\pm \frac{IR_{s,i}}{3\varepsilon_{s,i}FAL_{i}},i=n,p\text{,}$$

where $R_{s,i}$ is the particle radius of electrode particle, $\varepsilon_{s,i}$ is the volume fraction of solid phase, *F* is the Faraday constant, *A* is the area of electrode, $L_{i}$ is the thickness of electrode, *n*, *p* represent negative and positive electrode respectively. When the cell is discharging, the current *I* is defined as negative (*I* < 0). Therefore, the lithium-ion pore wall flux that flowed into electrode particles is positive, and the lithium-ion pore wall flux that flowed out of electrode particles is negative.

The lithium-ion capacity of electrodes is related to the volume of electrodes and the maximum lithium-ion concentration of electrode material. Therefore, the lithium-ion capacity of electrodes can be calculated as^27^

(Equation 2) $$Q_{i}=AL_{i}F\varepsilon_{s,i}c_{s,i}^{\max},i=n,p\text{,}$$

where $Q_{i}$ is the electrode capacity and $c_{s,i}^{\max}$ is the maximum lithium-ion concentration of the electrode material.

The solid phase lithium-ion concentration stoichiometry of electrodes is defined as the ratio of the electrode solid phase lithium-ion concentration to the electrode maximum solid phase lithium-ion concentration. Therefore, the initial solid phase stoichiometry $\theta_{i}^{0}$ and the average solid phase stoichiometry $\theta_{i}^{ave}$ can be expressed as

(Equation 3) $$\theta_{i}^{0}=\frac{c_{s,i}^{0}}{c_{s,i}^{\max}},\theta_{i}^{ave}=\frac{c_{s,i}^{ave}}{c_{s,i}^{\max}}\text{,}$$

where $c_{s,i}^{0}$ is the initial solid phase lithium-ion concentration of electrodes, $c_{s,i}^{ave}$ is the average solid phase lithium-ion concentration of electrodes. The average solid phase stoichiometry can be calculated by the initial solid phase stoichiometry and coulomb counting, as shown in Equation 4.

(Equation 4) $$\theta_{i}^{ave}=\theta_{i}^{0}+\frac{1}{Q_{i}}\int_{0}^{t_{k}}I\left(t\right)dt\text{,}$$

where $t_{k}$ is the cell operation time.

The open circuit voltage is defined as the voltage between the positive and negative electrodes when the cell reaches an equilibrium state, which can be expressed as

(Equation 5) $$U_{ocv}=U_{p}\left(\theta_{p}^{ave}\right)-U_{n}\left(\theta_{n}^{ave}\right)\text{,}$$

where $U_{p}\left(\cdot \right)$, $U_{n}\left(\cdot \right)$ are the positive and negative potentials as a function of positive and negative solid phase stoichiometry, respectively.

##### Solid phase diffusion overpotential

The solid phase diffusion overpotential is caused by the uneven distribution of lithium ions in electrode particles due to lithium-ion diffusion when the cell charges and discharges.^28^ The lithium-ion diffusion in electrode particles is governed by Fick’s second law as shown in Equation 6.

(Equation 6) $$\frac{\partial c_{s,i}\left(r,t\right)}{\partial t}=\frac{D_{s,i}}{r^{2}}\frac{\partial }{\partial r}\left(r^{2}\frac{\partial c_{s,i}\left(r,t\right)}{\partial r}\right),0\leq r\leq R_{s,i}\text{,}$$

where $c_{s,i}\left(r,t\right)$ is the solid phase lithium-ion concentration, $D_{s,i}$ is the solid phase diffusion coefficient, $R_{s,i}$ is the electrode particle radius. The boundary conditions of Equation 6 are

(Equation 7) $$D_{s,i}\frac{\partial c_{s,i}\left(0,t\right)}{\partial r}=0,D_{s,i}\frac{\partial c_{s,i}\left(R_{s,i},t\right)}{\partial r}=J_{i}\left(t\right)\text{.}$$

Perform Laplace transform on Equation 6 and combine its boundary conditions Equation 7, the relationship between the electrode surface lithium-ion concentration change relative to the initial electrode lithium-ion concentration ${\tilde{c}}_{s,i}^{surf}=c_{s,i}^{surf}-c_{s,i}^{0}$ and the lithium-ion pore wall flux $J_{i}$ can be derived as^29^

(Equation 8) $$\frac{{\tilde{c}}_{s,i}^{surf}}{J_{i}}=\frac{R_{s,i}}{D_{s,i}}\left[\frac{1}{\sqrt{\frac{R_{s,i}^{2}}{D_{s,i}}s}\;\coth\left(\sqrt{\frac{R_{s,i}^{2}}{D_{s,i}}s}\right)-1}\right]\text{.}$$

where *s* is the Laplace differential operator. The solid phase diffusion time constant is defined as

(Equation 9) $$\tau_{i}=\frac{R_{s,i}^{2}}{D_{s,i}}\text{.}$$

However, the system is unstable, there is a pole at *s* = 0 in Equation 8. To establish a stable transfer function, the solid phase surface lithium-ion concentration change after removing the integrator pole is defined as $\Delta {\tilde{c}}_{s,i}^{surf}={\tilde{c}}_{s,i}^{surf}-c_{s,i}^{ave}$. The relationship between $c_{s,i}^{ave}$ and $J_{i}$ can be expressed as

(Equation 10) $$\frac{c_{s,i}^{ave}}{J_{i}}=-\frac{3}{R_{s,i}s}\text{.}$$

Therefore, the relationship between $\Delta {\tilde{c}}_{s,i}^{surf}$ and $J_{i}$ can be calculated as^29^

(Equation 11) $$\frac{\Delta {\tilde{c}}_{s,i}^{surf}}{J_{i}}=\frac{\tau_{i}}{R_{s,i}}\left[\frac{1}{\sqrt{\tau_{i}s}\;\coth\left(\sqrt{\tau_{i}s}\right)-1}-\frac{3}{\tau_{i}s}\right]\text{.}$$

Since the electrode particle radius and the solid-phase diffusion coefficient are constant, the key dynamics of $\frac{\Delta {\tilde{c}}_{s,i}^{surf}}{J_{i}}$ can be extracted from Equation 11 as an intermediate transform function H(s).

(Equation 12) $$H\left(s\right)=\frac{1}{\sqrt{\tau_{i}s}\;\coth\left(\sqrt{\tau_{i}s}\right)-1}-\frac{3}{\tau_{i}s}\text{.}$$

H(s) is a transcendental transfer function, which is still too complex to be applied in real-time embedded systems. To simplify Equation 12, the reduced-order methods will be introduced in the following section.

The electrode solid phase surface stoichiometry $\theta_{i}^{surf}$ can be calculated easily by $\Delta {\tilde{c}}_{s,i}^{surf}$ and $\theta_{i}^{ave}$ as

(Equation 13) $$\theta_{n}^{surf}=\theta_{n}^{ave}+\frac{\Delta {\tilde{c}}_{s,n}^{surf}}{c_{s,n}^{\max}},\theta_{p}^{surf}=\theta_{p}^{ave}-\frac{\Delta {\tilde{c}}_{s,p}^{surf}}{c_{s,p}^{\max}}\text{.}$$

When the cell charges and discharges, solid phase diffusion causes an uneven lithium-ion distribution in the electrode particle, resulting in the electrode particle surface potential deviating from the equilibrium state.^30^ Therefore, the solid phase diffusion overpotential $\eta_{s}$ is defined as

(Equation 14) $$\eta_{s}=U_{p}\left(\theta_{p}^{surf}\right)-U_{n}\left(\theta_{n}^{surf}\right)-U_{ocv}\text{.}$$

##### Electrolyte phase diffusion overpotential

The lithium-ion diffusion in the electrolyte conforms to Maxwell-Stefan theory, which can be derived as

(Equation 15) $$\varepsilon_{e,i}\frac{\partial c_{e,i}\left(x,t\right)}{\partial t}=\frac{\partial }{\partial x}\left(D_{e,i}^{eff}\frac{\partial c_{e,i}\left(x,t\right)}{\partial x}\right)+a_{s,i}\left(1-t_{+}^{0}\right)J_{i}\text{,}$$

where $\varepsilon_{e,i}$ is the electrolyte phase volume faction. *x* ($0\leq x\leq L_{n}+L_{sep}+L_{p}$) is the location of cell thickness direction, where $L_{sep}$ represents the thickness of separator. $c_{e,i}\left(x,t\right)$ is the lithium-ion concentration in the electrolyte, which is related to time and location. $t_{+}^{0}$ is the lithium-ion transference number in the electrolyte. $D_{e,i}^{eff}$ is the effective electrolyte ionic diffusivity, which can be calculated as

(Equation 16) $$D_{e,i}^{eff}=D_{e}\varepsilon_{e,i}^{brugg}\text{,}$$

where $D_{e}$ is electrolyte ionic diffusivity, brugg is Bruggeman coefficient, the specific surface area of electrodes. $a_{s,i}$ is the specific surface area of electrodes, which is determined as

(Equation 17) $$a_{s,i}=\frac{3\varepsilon_{s,i}}{R_{s,i}}\text{.}$$

The lithium-ion concentration overpotential in the electrolyte is caused by the uneven electrolyte phase lithium-ion concentration distribution between positive and negative electrodes, which can be calculated as

(Equation 18) $$\eta_{e}=\left(1+t_{+}^{0}\right)\frac{2R_{g}T}{F}\ln\frac{c_{e,p}}{c_{e,n}}\text{,}$$

where $c_{e,p}$ and $c_{e,n}$ represent the lithium-ion concentration in positive and negative electrolyte phases at the terminal of the electrode near the current collector, respectively.

##### Lumped ohmic overpotential

The electrochemical reaction takes place on the surface of the electrode particles. The electrochemical reaction is a medium-high frequency dynamic, and the reaction time is usually in seconds.^31^ By linearizing the Butler-Volmer equation via Taylor-series expansion and decoupling the reaction flux *j* and the electrolyte concentration $c_{e,i}$, the electrochemical reaction impedance can be approximated by an equivalent resistance $R_{ct}$.

The lumped ohmic resistance contains the equivalent resistance of electrochemical reaction $R_{ct}$, solid-electrolyte interphase film resistance $R_{SEI}$, the resistance of solid and electrolyte material $R_{mat}$, and contact resistance $R_{con}$. Therefore, the lumped ohmic resistance can be calculated as

(Equation 19) $$R_{ohm}=R_{ct}+R_{SEI}+R_{mat}+R_{con}\text{.}$$

The lumped ohmic overpotential can be expressed as

(Equation 20) $$\eta_{ohm}=R_{ohm}I\text{.}$$

The cell terminal voltage is the sum of the open circuit voltage and all overpotentials, which is given as

(Equation 21) $$V_{cell}=U_{ocv}+\eta_{s}+\eta_{e}+\eta_{ohm}\text{.}$$

### Ensemble learning model

**Figure 2:**
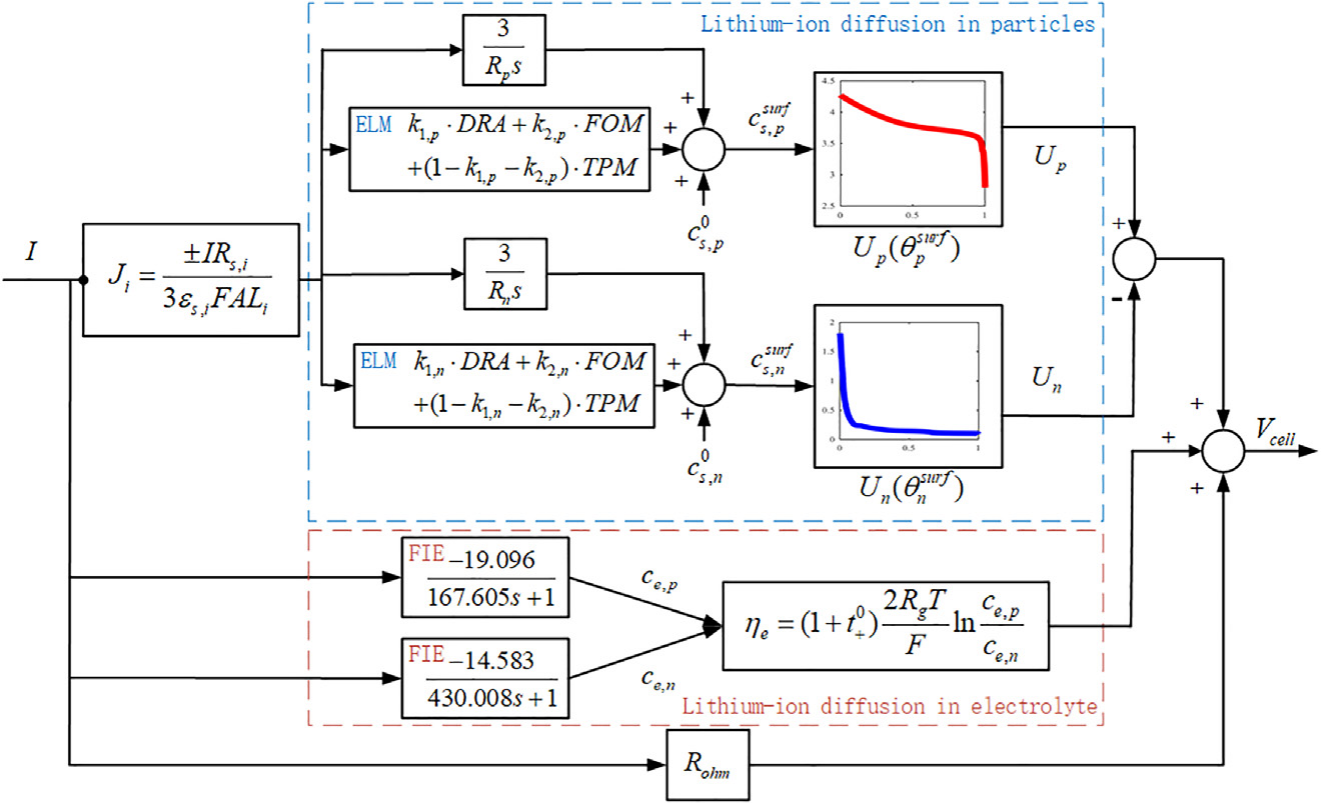
The structure of the simplified electrochemical model with ensemble learning

In order to accelerate the calculation speed of electrochemical models and accurately predict the internal states of lithium-ion batteries, a simplified electrochemical model with ensemble learning is proposed. The proposed model simplifies lithium-ion transfer in solid phase with ensemble learning model, and simplifies lithium-ion transfer in electrode phase with FIE. Finally, the cell voltage can be calculated according to the lithium-ion concentration in solid and electrolyte phases and the ohmic resistance. The structure of the proposed model is shown in Figure 2.

#### Reduced-order methods

According to the modeling process of the simplified electrochemical model, there is a transcendental transfer function in modeling the electrode particle surface lithium-ion concentration. To apply the simplified electrochemical model in real-time embedded systems, the transcendental transfer function should be simplified.^32^

##### Discrete-time realization algorithm

The DRA approximates the transcendental transfer function by a reduced-order discrete-time state-space realization, as the form of Equation 22.

(Equation 22) $$\begin{array}{c} x\left[k+1\right]=Ax\left[k\right]+Bu\left[k\right]\\ y\left[k\right]=Cx\left[k\right]+Du\left[k\right] \end{array}$$

The DRA is performed on the transcendental transfer function in the simplified electrochemical model. The continuous-time impulse response of the transcendental transfer function H(s) can be approximated by the frequency-domain emulation approach, as shown in Equation 23.

(Equation 23) $$H_{d}\left(z\right)\approx H\left(s\right){|}_{s=\frac{2}{T_{1}}\frac{z-1}{z+1}}$$

where $T_{1}$ is the emulation sampling period. The emulation sampling period $T_{1}$ is set to 1/256s, so that $1/T_{1}$ is much greater than the bandwidth of H(s). The discrete Fourier transform (DFT) of a sequence is related to its z-transform as

(Equation 24) $$H_{d}\left[f\right]=H_{d}\left(z\right){|}_{z=\exp\left(j2\pi f/N\right)}=H\left(\frac{2}{T_{1}}\left[\frac{e^{j2\pi f/N}-1}{e^{j2\pi f/N}+1}\right]\right),0\leq f\leq N$$

where *N* is the point number for the basic sequence, and is usually a power of 2 for efficient computation. By performing the inverse DFT to $H_{d}\left[f\right]$, the approximation of the continuous-time impulse response $h_{d}\left[n\right]$ at the emulation sampling period $T_{1}$ can be obtained as

(Equation 25) $$h_{d}\left[n\right]=\frac{1}{N}\sum_{f=0}^{N-1}H_{d}\left[f\right]e^{i2\pi fn/N}\text{.}$$

The cumulative summation of the continuous-time impulse response is the approximation of the continuous-time step response $h_{step}$ as

(Equation 26) $$h_{step}\left[k\right]=T_{1}\sum_{i=0}^{k-1}h_{d}\left[i\right]\text{.}$$

The discrete-time unit-pulse response h[k] can be calculated by the continuous-time step response as

(Equation 27) $$h\left[k\right]=h_{step}\left[k\right]-h_{step}\left[k-T_{s}\right]\text{,}$$

where $T_{s}$ is the inter-sample period.

The Ho-Kalman algorithm^33^ is used to find the state-space realization from the discrete-time unit-pulse response. The dimension of the reduced-order model is chosen to be 2 considering a tradeoff between complexity and accuracy. The parameters of the simulation cell are shown in Table 1^34^, the state-space realization matrices *A*, *B*, and *C* of the simulation cell are generated to approximate H(s) by the Ho-Kalman algorithm as

(Equation 28) $$A_{p}=\left[\begin{array}{cc} 0.8316 & 0.2408\\ 0.2408 & 0.4341 \end{array}\right],B_{p}=\left[\begin{array}{c} 1388.3\\ -1103.1 \end{array}\right]$$

$$C_{p}=\left[\begin{array}{cc} 1388.3 & -1103.1 \end{array}\right]\text{.}$$

(Equation 29) $$A_{n}=\left[\begin{array}{cc} 0.9710 & 0.0797\\ 0.0797 & 0.5939 \end{array}\right],B_{n}=\left[\begin{array}{c} 1218.3\\ -1820.9 \end{array}\right]$$

$$C_{n}=\left[\begin{array}{cc} 1218.3 & -1820.9 \end{array}\right]\text{.}$$

**Table 1:** Model parameters for a 17.5Ah $\mathrm{LiM}\;n_{2}O_{4}/Carbon$ cell

Parameters	Negative electrode	Separator	Positve electrode
L(m)	100e-6	52e-6	183e-6
$R_{s}\left(m\right)$	12.5e-6	–	8e-6
$\varepsilon_{s}$	0.471	–	0.297
$c_{s}^{\max}\left(mol/m^{3}\right)$	26390	–	22860
$c_{s}^{0}\left(mol/m^{3}\right)$	14870	–	3900
$D_{s}\left(m^{2}/s\right)$	3.9e-14	–	1e-13
$i_{0}\left(A/m^{2}\right)$	17.71	–	16.74
$c_{e}^{0}\left(mol/m^{3}\right)$		1000	
$D_{e}\left(m^{2}/s\right)$		7.5e-11	
$t_{+}^{0}$		0.363	
brugg		1.5	
T(K)		298.15	
F(C/mol)		96485.33	
$R_{g}$ (J/mol/K)		8.314	
$R_{ohm}$ (Ω)		0.0012	
A($m^{2}$)		1	
$U_{p}\left(\theta \right)$	4.19829+0.0565661tanh(−14.5546θ+8.60942)		
	$-0.0275479\left[\frac{1}{{\left(0.998432-\theta \right)}^{0.492465}}-1.90111\right]$		
	$-0.157123\;\exp\;\left(-0.04738\theta^{8}\right)+0.810239\;\exp\;\left[-40\left(\theta -0.133875\right)\right]$		
$U_{n}\left(\theta \right)$	−0.16 + 1.32exp(-3.0θ)+10.0exp(-2000.0θ)		

Because of the different constant time value $\tau_{i}$ of positive and negative electrodes, the reduced-order discrete-time state-space matrices of positive and negative electrodes are different. However, the $D_{i}$ matrix represents the high-frequency response of H(s), which can be calculated as $D_{i}=\lim_{s\to \infty }H\left(s\right)=0$. Therefore, the $D_{i}$ matrix of positive and negative electrodes are the same.

The DRA only requires the standard linear-algebra and signal-processing methods to realize the optimal reduced-order discrete-time approximation to the original continuous-time system, which does not need optimization and iteration. Therefore, the DRA has low computational complexity.

##### Fractional-order Padé approximation

The Padé approximation is a rational polynomial approximation method, which is widely applied in order reduction for complex systems and the solution of fractional-order equations.^35^ For an arbitrary function f(x), the Padé approximation form can be expressed as

(Equation 30) $$f\left(x\right)=\frac{a_{0}+a_{1}x+\cdots +a_{n}x^{n}}{1+b_{1}x+\cdots +b_{m}x^{m}}\text{,}$$

where *a* and *b* are the numerator and denominator polynomial coefficients, respectively. *n* and *m* are the order of numerator and denominator polynomials, respectively. Guo et al.^16^ have proven that the approximation of the transcendental function H(s) cannot be realized by simple low-order rational polynomials by analyzing the basic properties of logarithmic frequency characteristics. To simplify the approximation of H(s) and avoid too many polynomial coefficients to be identified, the basic order of Padé approximation form is set to be 1/2 according to the characteristics of H(s). Hence, the fractional-order Padé approximation form can be expressed as

(Equation 31) $$f\left(x\right)=\frac{a_{0}+a_{1}x^{\frac{1}{2}}+\cdots +a_{n}x^{\frac{1}{2}n}}{1+b_{1}x^{\frac{1}{2}}+\cdots +b_{m}x^{\frac{1}{2}m}}\text{.}$$

To achieve low-order fractional Padé approximation, *m* and *n* are set to 1 and 0, respectively. Therefore, the fractional-order Padé approximation form for the transcendental function H(s) can be expressed as

(Equation 32) $$H\left(s\right)=\frac{a_{0}}{1+b_{1}{\left(\tau s\right)}^{\frac{1}{2}}}$$

In accordance with Padé approximation theory,^36^ the coefficients $a_{0}$ and $b_{1}$ can be calculated as $a_{0}=1/5$ and $b_{1}=12/95$, respectively. Hence, the fractional-order Padé approximation function of the transcendental transfer function can be expressed as

(Equation 33) $$H\left(s\right)=\frac{19}{95+12{\left(\tau s\right)}^{\frac{1}{2}}}$$

##### Three-parameter parabolic approximation

The solid phase lithium-ion concentration distribution in the radius direction can be approximated by a three-parameter parabolic function as shown in Equation 34.

(Equation 34) $$c_{s,i}\left(r,t\right)=a\left(t\right)+b\left(t\right)\frac{r^{2}}{R_{i}^{2}}+c\left(t\right)\frac{r^{4}}{R_{i}^{4}},0\leq r\leq R\text{.}$$

where variables *a*, *b*, and *c* changes with time.

In accordance with the derivation of Subramanian et al.,^37^ when substituting Equation 34 into Equation 7 to solve variables *a*, *b* and *c*, the following equations can be obtained

(Equation 35) $$\frac{d}{dt}q_{i}^{ave}\left(t\right)+30\frac{D_{s,i}}{R_{i}^{2}}q_{i}^{ave}\left(t\right)-\frac{45}{2}\frac{J_{i}}{R_{i}^{2}}=0$$

(Equation 36) $$35\frac{D_{s,i}}{R_{i}}\left[{\tilde{c}}_{s,i}^{surf}\left(t\right)-c_{s,i}^{ave}\left(t\right)\right]-8D_{s,i}q_{i}^{ave}\left(t\right)=J_{i}$$

where $q_{i}^{ave}$ is the solid phase lithium-ion concentration per unit volume. Therefore, the relationship between $\Delta {\tilde{c}}_{s,i}^{surf}$ and the lithium-ion pore wall flux $J_{i}$ can be approximated by the following state-space functions

(Equation 37) $$\begin{array}{c} {\dot{q}}_{i}^{ave}=-\frac{30D_{s,i}}{R_{i}^{2}}q_{i}^{ave}+\frac{45}{2R_{i}^{2}}J_{i}\text{,}\\ \Delta {\tilde{c}}_{s,i}^{surf}=\frac{8R_{i}}{35}q_{i}^{ave}+\frac{R_{i}}{35D_{s,i}}J_{i}\text{.} \end{array}$$

The lithium-ion pore wall flux $J_{i}$ can be calculated by the applied current *I* according to Equation 1, then the transcendental transfer function H(s) can then be approximated by Equation 37.

#### Weighted averaging ensemble method

Ensemble learning is a typical machine learning technique that combines multiple models to achieve better predictive performance than any of the individual models alone. Error-ambiguity decomposition is a mathematical method for analyzing the performance of ensemble methods,^38^ which considers the influence of the average error of individual models and the average ambiguity of individual models. The error-ambiguity decomposition is shown in Equation 38.

(Equation 38) $$E=\bar{E}-\bar{A}$$

where $\bar{E}$ is the average error of individual models, $\bar{A}$ is the average ambiguity of individual models, *E* is the prediction error of ensemble model. The error-ambiguity decomposition theory shows that the more accurate and diverse the individual models, the better the ensemble.

An ensemble learning model (ELM), which simplifies the calculation of electrode particle surface lithium-ion concentration, is proposed to improve the prediction accuracy of lithium-ion mass transfer in electrode particles. The DRA, FOM, and TPM have shown certain accuracy in the prediction of electrode particle surface lithium-ion concentration in previous literature.^16^^,^^33^^,^^37^ There are large differences among DRA, FOM, and TPM according to the derivation in reduced-order methods section. The ensemble of DRA, FOM, and TPM can achieve better predictive performance in accordance with the error-ambiguity decomposition theory. The proposed ELM ensembles DRA, FOM, and TPM with the weighted average method. The ELM outputs are defined as

(Equation 39) $$ELM=k_{1}*DRA+k_{2}*FOM+\left(1-k_{1}-k_{2}\right)*TPM$$

where $k_{1}$ and $k_{2}$ are the weighted coefficients of the ensemble learning model.

#### Electrolyte phase diffusion simplification

To reduce the computational complexity of the lithium-ion distribution in the electrolyte, a FIE is proposed to simplify the lithium-ion diffusion in the electrolyte. The lithium-ion concentration in the electrolyte near the current collector directly affects the cell voltage. Therefore, the proposed FIE is used to fit the lithium-ion concentration change in the electrolyte near the current collector $\Delta c_{e,i}=c_{e,i}-c_{e,0}$, which can be expressed as

(Equation 40) $$\frac{\Delta c_{e,i}}{I}=\frac{K_{e}}{T_{e}s+1}$$

where $c_{e,0}$ is the initial lithium-ion concentration in the electrolyte, $K_{e}$ is the gain of electrolyte phase diffusion, $T_{e}$ is the time constant of electrolyte phase diffusion.

### Simulation and model verification

#### Weighted coefficient identification of ELM

Although the ensemble learning model is specifically designed for predicting the electrode particle surface lithium-ion concentration, the weighted coefficients are still unknown. A 17.5Ah $\mathrm{LiM}\;n_{2}O_{4}/Carbon$ cell is used for the weighted coefficients identification of ELM and model simulation, and its electrochemical parameters are shown in Table 1.

**Figure 3:**
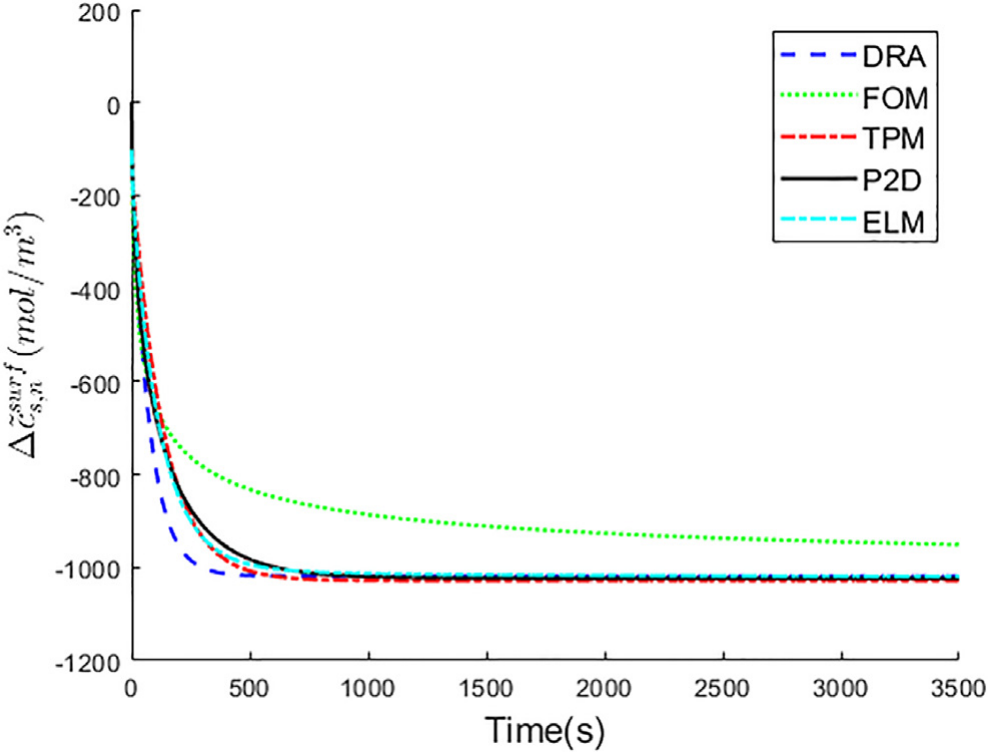
$\Delta {\tilde{c}}_{s,n}^{surf}$ comparison among DRA, FOM, TPM, ELM, and P2D

The particle swarm optimization (PSO) algorithm is used for identifying the weighted coefficients of ELM. The loss function is defined as the root-mean-square error (RMSE) between the output of ELM and the ground truth $\Delta {\tilde{c}}_{s,i}^{surf}$, shown in Equation 41.

(Equation 41) $$Loss=\sqrt{\frac{1}{n}\sum_{k=1}^{n}{\left(\Delta {\tilde{c}}_{s,k}^{surf}-\Delta {\tilde{c}}_{s,k,ELM}^{surf}\right)}^{2}}$$

where $\Delta {\tilde{c}}_{s,ELM}^{surf}$ is the output of ELM. The electrode particle surface lithium-ion concentration changes after removing the integrator pole $\Delta {\tilde{c}}_{s}^{surf}$ of P2D model are taken as ground truth. The optimal weighted coefficients of ELM for the negative electrode are $k_{1,n}=0.2165$ and $k_{2,n}=0.0933$, respectively. The negative particle surface lithium-ion concentration changes after removing the integrator pole $\Delta {\tilde{c}}_{s,n}^{surf}$ comparison among DRA, FOM, TPM, ELM, and P2D is shown in Figure 3, and the predictive errors of DRA, FOM, TPM, and ELM are shown in Table 2.

**Table 2:** $\Delta {\tilde{c}}_{s,n}^{surf}$ error analysis among DRA, FOM, TPM, and ELM

	DRA	FOM	TPM	ELM
RMSE($mol/m^{3}$)	34.14	106.16	18.57	11.51
MAPE(%)	1.91	10.41	3.84	2.63

In Figure 3, the $\Delta {\tilde{c}}_{s,n}^{surf}$ curve of ELM is significantly closer to that of the P2D model, which represents ELM achieves best predictive accuracy on $\Delta {\tilde{c}}_{s,n}^{surf}$. The same result is shown in Table 2. ELM gets the lowest $\Delta {\tilde{c}}_{s,n}^{surf}$ RMSE with only 11.51 $mol/m^{3}$. TPM is more accurate than DRA and FOM. Therefore, the weighted coefficient of TPM is larger than that of DRA and FOM in ELM.

**Figure 4:**
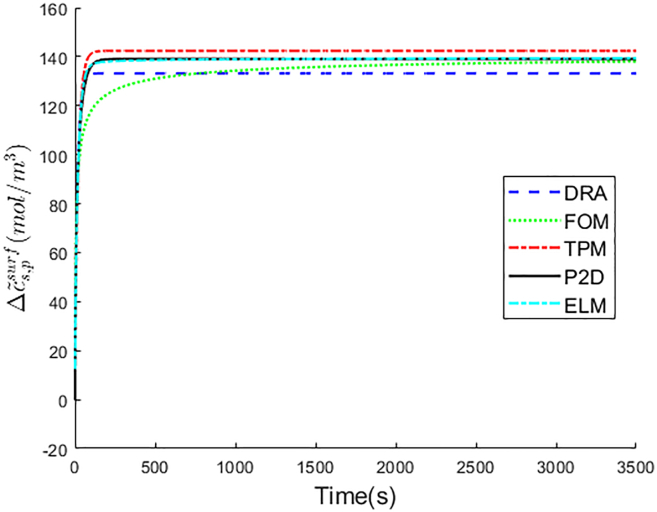
$\Delta {\tilde{c}}_{s,p}^{surf}$ comparison among DRA, FOM, TPM, ELM, and P2D

The optimal weighted coefficients of ELM for the positive electrode are $k_{1,p}=0.2867$ and $k_{2,p}=0.0998$, respectively. The $\Delta {\tilde{c}}_{s,p}^{surf}$ of DRA, FOM, TPM, ELM, and P2D are compared in Figure 4, and the $\Delta {\tilde{c}}_{s,p}^{surf}$ error analysis among DRA, FOM, TPM, and ELM is shown in Table 3.

**Table 3:** $\Delta {\tilde{c}}_{s,p}^{surf}$ error analysis among DRA, FOM, TPM, and ELM

	DRA	FOM	TPM	ELM
RMSE($mol/m^{3}$)	5.79	5.79	3.44	0.6
MAPE(%)	4.17	3.17	4.8	1.66

In Figure 4, ELM shows the best $\Delta {\tilde{c}}_{s,p}^{surf}$ predictive performance, and the $\Delta {\tilde{c}}_{s,p}^{surf}$ curve of ELM closely follows that of P2D model. The $\Delta {\tilde{c}}_{s,p}^{surf}$ prediction error of ELM is minimal with only 0.6 $mol/m^{3}$ RMSE and 1.66% MAPE, while DRA, FOM, and TPM all show more prediction error.

### Parameter identification of FIE

Due to different parameters in positive and negative electrolytes, the lithium-ion concentrations in positive and negative electrolytes are different. Therefore, the parameters of FIE in positive and negative electrolytes need to be identified respectively. The PSO algorithm is used for identifying the parameters of FIE, and the loss function is defined as the RMSE between the output of FIE and the ground truth of electrolyte phase lithium-ion concentration near the current collector, shown in Equation 42

(Equation 42) $$Loss=\sqrt{\frac{1}{n}\sum_{k=1}^{n}{\left(c_{e,i}-c_{e,i,FIE}\right)}^{2}}$$

where $c_{e,i}$ is the ground truth of lithium-ion concentration in the electrolyte near the current collector, $c_{e,i,FIE}$ represents the prediction lithium-ion concentration of FIE.

**Figure 5:**
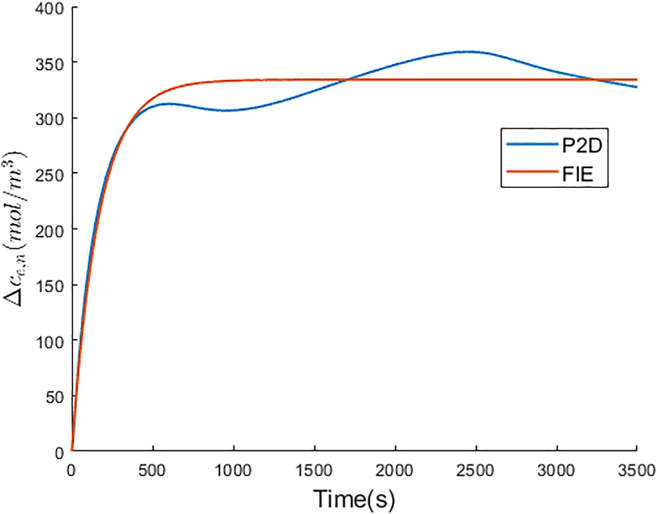
$\Delta c_{e,n}$ comparison between P2D and FIE

The optimal $K_{e}$ and $T_{e}$ of FIE for the negative electrolyte are −19.096 and 167.605, respectively. The $\Delta c_{e,n}$ of FIE is compared with that of P2D model, and is shown in Figure 5. In P2D model, due to the inhomogeneous lithium-ion flux on the electrode particle surface along the electrode thickness direction, the $\Delta c_{e,n}$ curve fluctuates after the relaxation time. In accordance with Assumption 3 in model structure section of the proposed simplified electrochemical model, the lithium-ion flux on the electrode particle surface is constant with constant current. Therefore, the $\Delta c_{e,n}$ of the proposed model remains constant after the relaxation time. In Figure 5, the $\Delta c_{e,n}$ curve of FIE is close to that of P2D model. FIE achieves 15.684 $mol/m^{3}$ RMSE for $\Delta c_{e,n}$ compared with P2D model.

**Figure 6:**
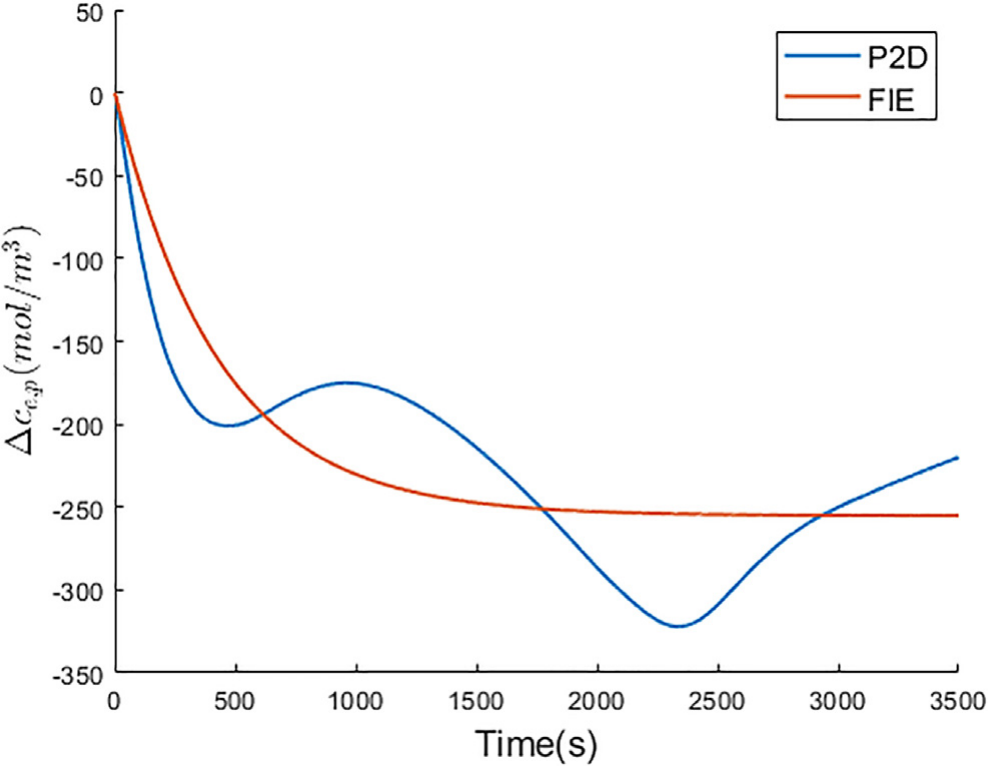
$\Delta c_{e,p}$ comparison between P2D and FIE

The optimal $K_{e}$ and $T_{e}$ of FIE for the positive electrolyte are 14.583 and 430.008, respectively. The $\Delta c_{e,p}$ comparison between FIE and P2D model is shown in Figure 6. In P2D model, the lithium-ion flux on the positive electrode particle surface is more unevenly distributed than that on the negative electrode particle surface because of $\epsilon_{p}<\epsilon_{n}$ and $L_{p}>L_{n}$. Therefore, the amplitude of $\Delta c_{e,p}$ fluctuations is greater than that of $\Delta c_{e,n}$ fluctuations in P2D model. FIE also achieves accurate $\Delta c_{e,p}$ prediction with 39.136 $mol/m^{3}$ RMSE compared with P2D model.

#### The cell voltage

**Figure 7:**
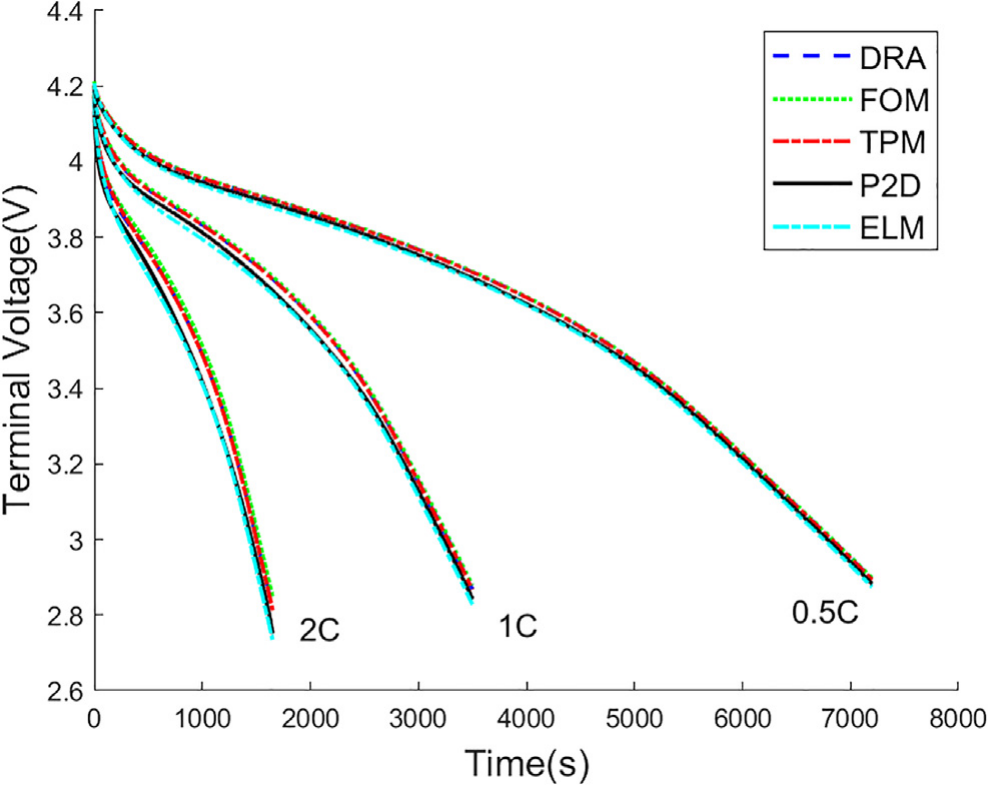
Voltage comparison among DRA, FOM, TPM, P2D, and ELM

The $\mathrm{LiM}\;n_{2}O_{4}/Carbon$ cell is simulated under 0.5C, 1C, and 2C rate constant current discharge, respectively. In order to validate the accuracy of the proposed ELM, P2D, DRA, FOM, and TPM are added for comparison. The voltage comparison among these models is shown in Figure 7. The voltage errors of ELM, DRA, FOM, TPM compared to P2D model are shown in Table 4. The voltage curves of DRA, FOM, TPM, and ELM are all very close to P2D model at 0.5C rate discharge, but ELM achieves the best voltage prediction with only 7.781mV RMSE and 0.209% MAPE. As the discharge rate increases, the voltage error of DRA, FOM, TPM, and ELM increases. Although the voltage curves of DRA, FOM, and TPM deviates significantly from that of P2D model at 1C and 2C discharge rate, the ELM voltage curve is still close to the P2D voltage curve. The results show that the proposed ELM achieves better voltage prediction over wide current range. Even if the cell is discharged at 2C current rate, the proposed ELM still achieves 37.71 mV RMSE and 0.985% MAPE.

**Table 4:** Voltage error analysis among DRA, FOM, TPM, and ELM

Discharge	DRA		FOM		TPM		ELM	
Rate	RMSE(mV)	MAPE(%)	RMSE(mV)	MAPE(%)	RMSE(mV)	MAPE(%)	RMSE(mV)	MAPE(%)
0.5C	12.57	0.347	14.96	0.415	12.21	0.336	7.781	0.209
1C	45.35	1.219	52.11	1.425	44.62	1.204	14.08	0.324
2C	72.78	2.084	93.82	2.701	72.75	2.093	37.71	0.985

To compare the computational complexity of DRA, FOM, TPM, ELM, and P2D model, all these models are simulated at 1C rate constant current discharge in Windows 11 with Intel(R) Core(TM) i7-10700F CPU @2.90 Ghz, 32G RAM. The computational time of these models is shown in Table 5. The computational speeds of DRA and TPM are faster because there are only two second-order state-space equations in DRA and TPM. FOM is slightly slower due to the fractional-order transfer function. ELM only takes 0.1676s to complete the 3500s 1C constant current discharge. The calculation speed of the P2D model is much slower than that of the other models. On the one hand, accurate calculation of electrode surface lithium-ion concentration is more complicated, which costs more computational time. On the other hand, the calculation of electrolyte phase and solid phase potential distribution along the cell thickness direction costs extra computational time.

**Table 5:** Computational time of DRA, FOM, TPM, ELM, and P2D

Battery models	Computational time
DRA	0.0201s
FOM	0.1271s
TPM	0.0139s
ELM	0.1676s
P2D	1min12s

**Figure 8:**
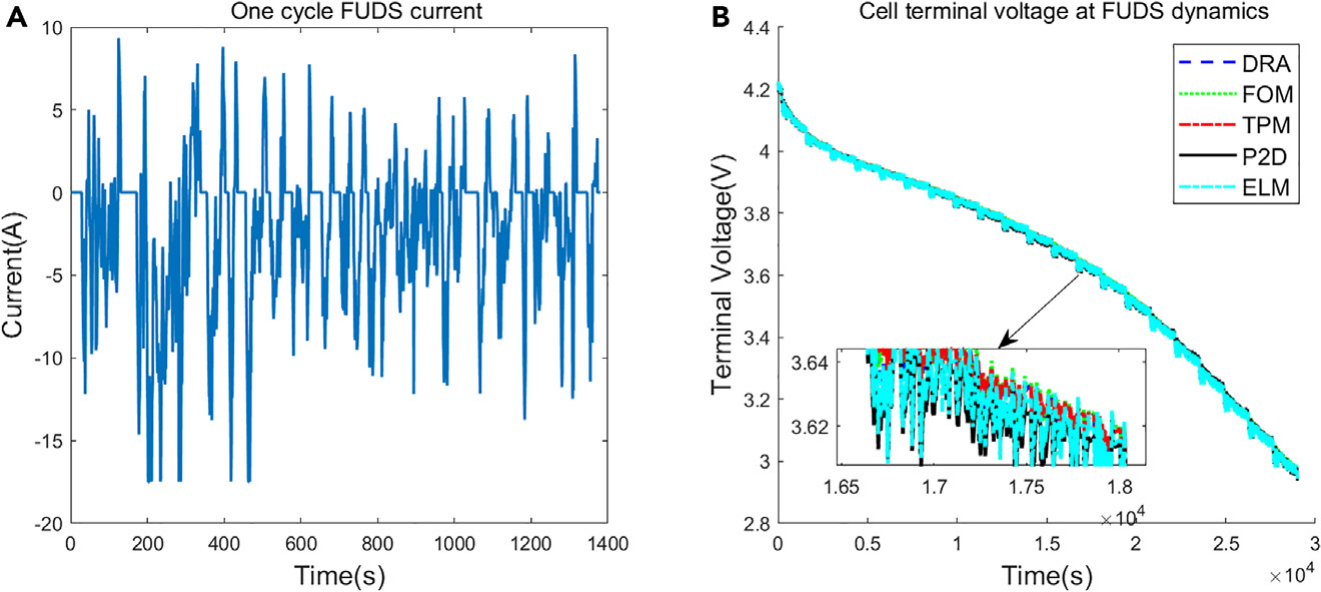
Simulation at FUDS dynamics (A) One cycle FUDS current. (B) Cell voltage at FUDS dynamics.

To further validate the effectiveness of the proposed model under dynamic operating conditions, DRA, FOM, TPM, ELM, and P2D are simulated at the federal urban driving schedule (FUDS) dynamics. FUDS dynamic takes 1372s to complete one full cycle, the cell is simulated with multiple FUDS cycles until the cell reaches the cut-off voltage. The simulation current and voltage are shown in Figure 8, and the voltage errors of these models are shown in Table 6. Since the current rate is small in most of time of FUDS dynamics, the voltage curves of DRA, FOM, TPM, and ELM are all close to that of P2D model. However, ELM still achieves the most accurate voltage prediction among these models with only 4.48 mV RMSE and 0097% MAPE.

**Table 6:** Voltage errors at FUDS dynamics

	DRA	FOM	TPM	ELM
RMSE(mV)	9.16	10.17	10.43	4.48
MAPE(%)	0.210	0.244	0.242	0.097

### Conclusions

The mass transfer in lithium-ion batteries not only determines the electrode potential but also influences the electrochemical reaction rates. It is critical for an electrochemical model to achieve accurate lithium-ion transfer prediction in electrodes. A simplified electrochemical model, which quickly and accurately characterizes lithium-ion transfer in lithium-ion batteries, is urgently needed for real-time embedded systems. Therefore, a simplified electrochemical lithium-ion batteries model with ensemble learning is proposed. The proposed model simplifies lithium-ion transfer in electrode particles by ensembling DRA, FOM, and TPM, which achieves precise lithium-ion concentration change prediction on the electrode particle surface. The proposed model simplifies lithium-ion transfer in the electrolyte with the FIE, which precisely predicts the lithium-ion concentration in the electrolyte attached to the current collector. The proposed ELM achieves more accurate voltage predictions than DRA, FOM, and TPM under both constant current and dynamic conditions. Although the proposed ELM introduces some parameters to be identified and takes a slightly longer time to compute than DRA, FOM, and TPM, its computational complexity is much less than that of P2D model. The proposed ELM achieves accurate solid-phase and electrolyte-phase lithium-ion concentration prediction and voltage prediction with low computational complexity, which provides a strong technical support for achieving future intelligent, multi-state volume monitoring BMSs.

## STAR★Methods

### Key resources table

**Table:** 

REAGENT or RESOURCE	SOURCE	IDENTIFIER
**Other**		

Negative electrode	Petroleum coke	https://doi.org/10.1149/1.1836921
Electrolyte	LiPF6 in 1:2 EC:DMC and p(VdF-HFP)	https://doi.org/10.1149/1.1836921
Positive electrode	LiMn_2_O_4_ Spinel	https://doi.org/10.1149/1.1836921

**Sotfware**		

Matlab	MathWorks	https://www.mathworks.com

### Resource availability

#### Lead contact

Further information and requests for resources and reagents should be directed to and will be fulfilled by the Lead Contact, Jing V Wang (jingvwang@whut.edu.cn).

#### Materials availability

This study did not generate new unique reagents.

#### Data and code availability

Data is available with no request, see more detail in the github https://github.com/kon9chun/SEMwithEL.

### Experimental model and subject details

The battery adopted in this investigation is a lithium-ion battery with LiMn_2_O_4_ (LMO) as the cathode and petroleum coke as the anode. The nominal capacity of the battery is 17.5Ah.

### Method details

The proposed ELM simplifies lithium-ion transfer in electrode particles by ensembling DRA, FOM, and TPM.

$$\text{ELM}=k_{1}*\text{DRA}+k_{2}*\text{FOM}+\left(1-k_{1}-k_{2}\right)*\text{TPM}$$

where $k_{1}$ and $k_{2}$ are the weighted coefficients of the ensemble learning model.

ELM simplifies the lithium-ion diffusion in the electrolyte with a first-order inertial element.

$$\frac{\Delta c_{e,i}}{I}=\frac{K_{e}}{T_{e}s+1}$$

where $c_{e,0}$ is the initial lithium-ion concentration in the electrolyte, $K_{e}$ is the gain of electrolyte phase diffusion, $T_{e}$ is the time constant of electrolyte phase diffusion.

### Quantification and statistical analysis

The simulation was run with matlab in Windows 11 with Intel(R) Core(TM) i7-10700F CPU @2.90 Ghz, 32G RAM. Figures are produced by Matlab from raw data.

### Additional resources

The data and source code are available with no request, see more detail in the github https://github.com/kon9chun/SEMwithEL.

## References

[1] Harper G., Sommerville R., Kendrick E., Driscoll L., Slater P., Stolkin R., Walton A., Christensen P., Heidrich O., Lambert S. (2019). Recycling lithium-ion batteries from electric vehicles. Nature.

[2] Yuan H., Zhu S., Akehurst S., Wang L., Wang L. (2021). A novel numerical implementation of electrochemical-thermal battery model for electrified powertrains with conserved spherical diffusion and high efficiency. Int. J. Heat Mass Tran..

[3] Zhu G., Kong C., Wang J.V., Kang J., Yang G., Wang Q. (2023). A fractional-order model of lithium-ion battery considering polarization in electrolyte and thermal effect. Electrochim. Acta.

[4] Plett G.L. (2004). Extended kalman filtering for battery management systems of lipb-based hev battery packs part 1. background. J. Power Sources.

[5] Plett G.L. (2004). Extended kalman filtering for battery management systems of lipb-based hev battery packs: Part 2. modeling and identification. J. Power Sources.

[6] Plett G.L. (2004). Extended kalman filtering for battery management systems of lipb-based hev battery packs: Part 3. state and parameter estimation. J. Power Sources.

[7] Doyle M., Fuller T.F., Newman J. (1993). Modeling of galvanostatic charge and discharge of the lithium/polymer/insertion cell. J. Electrochem. Soc..

[8] Fuller T.F., Doyle M., Newman J. (1994). Simulation and optimization of the dual lithium ion insertion cell. J. Electrochem. Soc..

[9] He C., Yue Q., Wu M., Chen Q., Zhao T. (2021). A 3d electrochemical-thermal coupled model for electrochemical and thermal analysis of pouch-type lithium-ion batteries. Int. J. Heat Mass Tran..

[10] Yu H., Zhang L., Wang W., Yang K., Zhang Z., Liang X., Chen S., Yang S., Li J., Liu X. (2023). Lithium-ion battery multi-scale modeling coupled with simplified electrochemical model and kinetic monte carlo model. iScience.

[11] Santhanagopalan S., Guo Q., Ramadass P., White R.E. (2006). Review of models for predicting the cycling performance of lithium ion batteries. J. Power Sources.

[12] Li X., Zhang Z., Gong L., Zhang Z., Liu G., Tan P. (2023). Revealing the mechanism of stress rebound during discharging in lithium-ion batteries. J. Energy Storage.

[13] Han X., Ouyang M., Lu L., Li J. (2015). Simplification of physics-based electrochemical model for lithium ion battery on electric vehicle. part i: Diffusion simplification and single particle model. J. Power Sources.

[14] Han X., Ouyang M., Lu L., Li J. (2015). Simplification of physics-based electrochemical model for lithium ion battery on electric vehicle. part ii: Pseudo-two-dimensional model simplification and state of charge estimation. J. Power Sources.

[15] Lee J.L., Chemistruck A., Plett G.L. (2012). Discrete-time realization of transcendental impedance models, with application to modeling spherical solid diffusion. J. Power Sources.

[16] Guo D., Yang G., Feng X., Han X., Lu L., Ouyang M. (2020). Physics-based fractional-order model with simplified solid phase diffusion of lithium-ion battery. J. Energy Storage.

[17] Luo W., Lyu C., Wang L., Zhang L. (2013). A new extension of physics-based single particle model for higher charge–discharge rates. J. Power Sources.

[18] Moura S.J., Argomedo F.B., Klein R., Mirtabatabaei A., Krstić M. (2017). Battery state estimation for a single particle model with electrolyte dynamics. IEEE Trans. Control Syst. Technol..

[19] Zhu G., Kong C., Wang J.V., Kang J., Wang Q., Qian C. (2023). A fractional-order electrochemical lithium-ion batteries model considering electrolyte polarization and aging mechanism for state of health estimation. J. Energy Storage.

[20] Shi J., Rivera A., Wu D. (2022). Battery health management using physics-informed machine learning: Online degradation modeling and remaining useful life prediction. Mech. Syst. Signal Process..

[21] Zhang Y., He X., Chen Z., Bai Q., Nolan A.M., Roberts C.A., Banerjee D., Matsunaga T., Mo Y., Ling C. (2019). Unsupervised discovery of solid-state lithium ion conductors. Nat. Commun..

[22] Cao M., Zhang T., Liu Y., Zhang Y., Wang Y., Li K. (2022). An ensemble learning prognostic method for capacity estimation of lithium-ion batteries based on the v-iowga operator. Energy.

[23] Zhang Y., Wang Y., Zhang C., Qiao X., Ge Y., Li X., Peng T., Nazir M.S. (2024). State-of-health estimation for lithium-ion battery via an evolutionary stacking ensemble learning paradigm of random vector functional link and active-state-tracking long–short-term memory neural network. Appl. Energy.

[24] Zhao X., Jung S., Wang B., Xuan D. (2023). State of charge estimation of lithium-ion battery based on improved adaptive boosting algorithm. J. Energy Storage.

[25] Ma C., Zhai X., Wang Z., Tian M., Yu Q., Liu L., Liu H., Wang H., Yang X. (2019). State of health prediction for lithium-ion batteries using multiple-view feature fusion and support vector regression ensemble. Int. J. Mach. Learn. Cybern..

[26] Martínez-Rosas E., Vasquez-Medrano R., Flores-Tlacuahuac A. (2011). Modeling and simulation of lithium-ion batteries. Comput. Chem. Eng..

[27] Li C., Cui N., Wang C., Zhang C. (2021). Simplified electrochemical lithium-ion battery model with variable solid-phase diffusion and parameter identification over wide temperature range. J. Power Sources.

[28] Zhang X., Li P., Huang B., Zhang H. (2022). Numerical investigation on the thermal behavior of cylindrical lithium-ion batteries based on the electrochemical-thermal coupling model. Int. J. Heat Mass Tran..

[29] Lee J.L., Chemistruck A., Plett G.L. (2012). One-dimensional physics-based reduced-order model of lithium-ion dynamics. J. Power Sources.

[30] Hamed H., Henderick L., Choobar B.G., D’Haen J., Detavernier C., Hardy A., Safari M. (2021). A limitation map of performance for porous electrodes in lithium-ion batteries. iScience.

[31] Jia Y., Dong L., Yang G., Jin F., Lu L., Guo D., Ouyang M. (2022). Parameter identification method for a fractional-order model of lithium-ion batteries considering electrolyte-phase diffusion. Batteries.

[32] Kawakita de Souza A., Hileman W., Trimboli M.S., Plett G.L. (2023). A control-oriented reduced-order model for lithium-metal batteries. IEEE Control Syst. Lett..

[33] Katayama T. (2005). http://link.springer.com/10.1007/1-84628-158-X.

[34] Doyle M., Newman J., Gozdz A.S., Schmutz C.N., Tarascon J. (1996). Comparison of modeling predictions with experimental data from plastic lithium ion cells. J. Electrochem. Soc..

[35] Basdevant J.L. (1972). The padé approximation and its physical applications. Protein Sci..

[36] Forman J.C., Bashash S., Stein J.L., Fathy H.K. (2011). Reduction of an electrochemistry-based li-ion battery model via quasi-linearization and padé approximation. J. Electrochem. Soc..

[37] Subramanian V.R., Diwakar V.D., Tapriyal D. (2005). Efficient macro-micro scale coupled modeling of batteries. J. Electrochem. Soc..

[38] Zhang Z., Dalca A.V., Sabuncu M.R. (2019). Confidence calibration for convolutional neural networks using structured dropout. ArXiv.

